# Progress on new drug treatments for hepatic echinococcosis

**DOI:** 10.3389/fphar.2026.1743435

**Published:** 2026-01-27

**Authors:** Yunzhang Li, Nana Xie, Jinping Chai, Qindong Jing, Linxun Liu, Jide A, Hongshuai Pan

**Affiliations:** 1 Clinical Medical College of Qinghai University, Xining, China; 2 Department of Internal Medicine-Cardiovascular, Qinghai Provincial People’s Hospital, Xining, China; 3 Department of General Surgery, Qinghai Provincial People’s Hospital, Xining, China

**Keywords:** albendazole, antitumor drugs, hepatic echinococcosis, Tibetan medicine, traditional Chinese medicine

## Abstract

*Echinococcosis*: is a zoonotic parasitic disease caused by infection with the larvae of *Echinococcus* tapeworms. At present, the standardized treatment regimen for this disease consists of radical lesion resection combined with albendazole. For patients who are inoperable or experience postoperative recurrence, the treatment with anti-echinococcosis drugs is particularly crucial. To date, albendazole remains the primary drug clinically used for anti-echinococcosis treatment. However, it has inherent drawbacks such as low bioavailability, poor solubility, long treatment course, and multiple adverse reactions, which reduce the medication adherence of patients with echinococcosis and compromise the clinical therapeutic efficacy. In recent years, with the in-depth development of research in molecular biology, pharmacology, and natural medicines, significant progress has also been made in the field of anti-echinococcosis drugs. This article systematically reviews the research progress in the development of new anti-echinococcosis drugs from four aspects: structural modification and new formulations of benzimidazole drugs, targeted drug therapy, combined medication strategies, and exploration of natural medicines and active components of traditional Chinese medicines.

## 1 Introduction

*Echinococcosis*, also known as hydatid disease, is a zoonotic parasitic disease caused by infection with the larvae of *Echinococcus* tapeworms. It mainly parasitizes in the liver, and secondarily affects other organs such as the lungs, brain, and bones (Liu et al., 2017). In clinical practice, the common pathological types include *Cystic Echinococcosis (CE)* caused by *Echinococcus granulosus (E.g)* and *Alveolar Echinococcosis (AE)* induced by *Echinococcus multilocularis* (E.m) (Liu et al., 2016). Up to now, echinococcosis has been detected in 23 provinces, municipalities and regions in China, among which the epidemic is relatively severe in the western pastoral and agricultural areas such as Qinghai, Xinjiang, Tibet, Gansu and Sichuan (Li et al., 2009). Radical lesion resection is the standard treatment for echinococcosis. However, due to its high surgical risk and high requirements for surgeons’ surgical skills, there are certain difficulties in promoting this technique. In addition, some echinococcosis patients have problems such as insidious disease course, late first diagnosis and large lesion volume, which make it difficult to perform radical lesion resection. For such patients, the treatment mainly relies on anti-echinococcosis drugs (Chen et al., 2023). Benzimidazoles, as the first-line drugs for the treatment of echinococcosis, have a long history of clinical application and a wide range of use. Currently, they remain an indispensable part of the echinococcosis prevention and control strategy, but they are associated with low efficacy and numerous adverse drug reactions (Zhu et al., 2017). It has also become a focus in clinical pharmacology, which has motivated drug researchers to seek safer and more effective anti-hydatid disease drugs. In recent years, with the continuous in-depth development of pharmaceutical chemistry, molecular biology, nanotechnology, and natural medicine research, the drug treatment strategies for echinococcosis have shown a developing trend of diversification and precision, which provides new ideas and directions for the research and development of anti-echinococcosis drugs. This article aims to systematically review the research progress in the drug treatment of echinococcosis in recent years, and summarize the relevant studies from multiple dimensions, including drug structure optimization, dosage form improvement, combined therapy, and exploration of natural medicines and traditional Chinese medicine components. It is expected to provide a theoretical basis and practical reference for the optimization of clinical treatment regimens and the research and development of new drugs.

## 2 The current research status of traditional anti-echinococcosis drugs

Albendazole, as a representative of benzimidazole class anti-parasitic drugs, works primarily by selectively inhibiting the polymerization of parasite microtubule proteins, interfering with glucose uptake and energy metabolism, thereby achieving an eradication effect on echinococciosis. As a representative of benzimidazole antiparasitic drugs, albendazole exerts its effect mainly by selectively inhibiting the polymerization of parasitic tubulin, interfering with glucose uptake and energy metabolism, thereby achieving the killing effect on echinococciosis (Wen et al., 2015). Huang et al. (2021) showed through animal experiments that albendazole liposomes can significantly increase drug accumulation in lesions by enhancing liver targeting, and their sustained-release properties allow the blood drug concentration to be maintained within the therapeutic window for a longer period. The team’s clinical observation on end-stage hepatic alveolar echinococcosis in Qinghai area revealed that albendazole monotherapy is difficult to effectively control the invasion of the hepatic portal and vena cava by the lesions, reflecting the limitations of existing drugs in advanced cases. Li WX. et al. (2024) pointed out that the long-term and single use of benzimidazole drugs may lead to drug resistance in fish parasites, a phenomenon similar to that observed in human echinococcosis. This finding provides a cross-species reference for the research on the drug resistance mechanism of echinococcosis. As the only drug currently applicable to both conditions, albendazole still holds an irreplaceable fundamental position.

Albendazole is a first-line anti-echinococcosis drug recommended by the World Health Organization (WHO) and widely used in clinical practice (Solomon et al., 2017). However, this drug has problems such as poor water solubility, unsatisfactory oral absorption, the need for long-term administration, and obvious side effects, which seriously affect its therapeutic effect. Therefore, the development of new and highly effective chemotherapeutic drugs is urgent.

## 3 Progress in the research and development of new drug dosage forms

### 3.1 Albendazole-bile acid derivative (ABZ-BA)

Bile acids are important components secreted by the liver. They play a key role in lipid metabolism and intestinal absorption, and can be actively absorbed in the terminal ileum through the Apical Sodium-Dependent Bile Acid Transporter (ASBT) (Deng and Bae, 2020; Saveleva et al., 2020). Gao et al. (2021) found through rat experiments that the expression of ASBT was upregulated in the ileum of model rats with hepatic alveolar echinococcosis. This finding suggests that the bile acid structure can be introduced into the albendazole molecule to derive albendazole-bile acid derivatives (ABZ-BA), thereby enhancing its transmembrane transport and oral bioavailability. Hu et al. (2023) further proposed a hypothesis: the steric hindrance effect of bile acids can reduce the crystallinity of the drug, thereby improving its solubility; and the specific transport mechanism of bile acids through ASBT can enhance the intestinal absorption efficiency of albendazole. The team successfully synthesized ABZ-BA and confirmed its structure using techniques such as mass spectrometry and nuclear magnetic resonance. Rat experiments showed that the equilibrium solubility of ABZ-BA was 4 times higher than that of albendazole, its relative bioavailability was 26 times higher, and it exhibited better *in vivo* pharmacokinetic performance. *In vitro* protoscolex inhibition experiments indicated that the anti-echinococcosis activity of ABZ-BA was comparable to that of albendazole, suggesting that structural modification did not affect its therapeutic efficacy. Pharmacodynamic results showed that the therapeutic efficacy of oral ABZ-BA after 1 month was 2.7 times that of the albendazole group. ABZ-BA has transformed from a crystalline state to an amorphous state, which significantly improves its bioavailability and demonstrates good therapeutic potential (Hu et al., 2023). However, its absorption, metabolism, and potential toxicity in the human body remain to be evaluated, and the differences in ASBT expression in the human body also require further research.

### 3.2 Nanoparticle delivery system

Nanotechnology shows broad prospects in targeted drug delivery and precision medicine. With the help of nanocarriers, chemotherapeutic drugs can achieve liver-targeted delivery, reduce damage to normal tissues, lower the risk of complications, and improve patients’ quality of life (Rafiei et al., 2019; Çolak et al., 2019; Aminpour et al., 2019). In addition, nanobiosensors can detect cyst-related biomarkers of echinococcosis with high sensitivity and specificity, thereby improving the accuracy of diagnosis (Jain et al., 2021). Huang et al. (2021) confirmed through animal experiments that albendazole nanospheres can passively target the hepatic sinusoids through particle size regulation (100–200 nm). The drug concentration of these nanospheres is 4.7 times higher than that of traditional preparations, and their sustained-release property prolongs the duration of effective blood drug concentration to more than 72 h. This study corresponds to the mechanism of transmembrane delivery of nanomedicines reported by Fan et al. (2024), that is, nanoparticles can penetrate the fibrotic barrier of hydatid cyst walls through the enhanced permeability and retention effect. The research team led by Liu et al. (2024) developed a graphene oxide/silver nanocomposite, which exhibits dual sustained-release properties. It stabilizes drug molecules through π-π conjugation, providing a new research direction for the design of nanocarriers for echinococcosis. Zhai et al. (2025) proposed that studies on histone deacetylase inhibitors further revealed that surface modification of nanocarriers with hepatocyte-specific ligands (such as galactose residues) can actively enhance liver uptake efficiency. This strategy increases the targeting efficiency to 2.3 times that of passive targeting systems. The Proteolysis Targeting Chimeras (PROTAC) molecular technology developed by Wu and Hu (2024) endows the nano-sustained release system with dynamic regulation capabilities. It can realize real-time feedback regulation of drug concentration through the ubiquitin-proteasome pathway, and this technology is expected to solve the clinical problem of narrow dose window in the treatment of echinococcosis. However, Li Y. et al. (2024) research on lipopolysaccharide transporter inhibitors warns that the biocompatibility of nanomaterials still requires attention. Her team found that some polymer carriers may induce dose-dependent kidney injury, which suggests that the long-term safety of nanoformulations for echinococcosis needs to be strictly evaluated. In addition, issues such as the difficulty in the biodistribution of nanomaterials, their inherent toxic properties, and the potential health problems they may cause still require attention (Richard, 2018).

### 3.3 The synergistic therapeutic effect of combined preparations on *Echinococcosis*

Compound preparations have significantly enhanced the therapeutic effect on multi-organ echinococcosis infections through multi-target synergistic effects. The “old drug repurposing” strategy proposed by Zhang et al. (2024) in the study of giardiasis provides a medication idea for the design of combined preparations for echinococcosis. Specifically, by re-evaluating the pharmacological properties of existing drugs, a combined medication regimen targeting different developmental stages of *Echinococcus granulosus* can be developed. For example, Praziquantel, as a broad-spectrum antihelminthic drug, exhibits a unique synergistic mechanism in the combined treatment of echinococcosis (Soleymani et al., 2025). It increases the permeability of the parasite’s body membrane to calcium ions, leading to spasmodic paralysis of the parasite and thereby enhancing the penetration effect of albendazole. When used in combination with albendazole, it can exert a synergistic effect in pre-operative and post-operative chemotherapy, especially reducing the risk of abdominal dissemination and recurrence when cyst fluid leaks during surgery (Mohamed et al., 1998; Cobo et al., 1998). Yasawy et al. (1993) published the first clinical trial on the treatment of echinococcosis with albendazole combined with praziquantel. The results showed that the combined group had stronger activity in killing larvae and anti-cyst, and it was easier to achieve clinical cure or improvement. Yaojun Ma’s research on *Cryptosporidium* drug resistance showed that long-term single use of praziquantel may lead to overexpression of the parasite’s ATP-binding cassette (ABC) transporters. This suggests that dynamic monitoring of praziquantel sensitivity changes is necessary in combined medication (Ma et al., 2025). Tang et al. (2024) further demonstrated through research that sequential administration of macrolides and praziquantel can reduce therapeutic toxicity. This finding provides a pharmacological basis for optimizing the administration timing of combined regimens for echinococcosis (Tang et al., 2024).

Furthermore, metformin exhibits antiproliferative effects in addition to its hypoglycemic action, potentially achieved by inducing cellular energy stress. The research team led by Loos et al. found that metformin exerts a dose- and time-dependent killing effect on protoscoleces and cysticerci of *Echinococcus granulosus in vitro*. After 3 and 12 days of combined use with albendazole sulfoxide, it showed a synergistic insecticidal effect. Oral administration of metformin can reduce the number and weight of cysts in infected mice, and the effect of combined medication is more significant, suggesting that metformin has potential value in the prevention and treatment of echinococcosis (Loos et al., 2022).

In summary, the combined use of albendazole with drugs such as praziquantel and metformin has shown potential for synergistic efficacy enhancement, and may overcome drug resistance through multi-target effects. However, most of the existing studies are limited to animal experiments, with small sample sizes and a lack of rigorous randomized controlled designs. High-quality clinical trials are urgently needed to verify their clinical application value. Research Progress on Novel Drug Formulations as Shown in Table 1.

**TABLE 1:** Advances in novel drug formulation development.

Drug category	Representative drugs	Main mechanism of action	*In vitro or* *in vivo*	Treatment effect
Albendazole-bile acid derivative	ABZ-BA	Reduce drug crystallinity, enhance intestinal absorption via ASBT; maintain anti-echinococcosis activity (Gao et al., 2021)	*In vitro+* *in vivo* (Hu et al., 2023)	Better therapeutic effect
Nanoparticle delivery system	Albendazole nanospheres (Huang et al., 2021)	Passive targeting to hepatic sinusoids via particle size regulation; penetrate cyst fibrotic barrier via EPR effect (Huang et al., 2021)	*In vivo*	Better therapeutic effect
Combined preparations	Albendazole + Praziquantel (Yasawy et al., 1993)	Praziquantel increases parasite membrane calcium permeability, induces spastic paralysis; enhances albendazole penetration (Soleymani et al., 2025).	Clinical trial, animal experiments	Better therapeutic effect
​	Albendazole sulfoxide + Metformin (Loos et al., 2022)	Metformin induces cellular energy stress, exerts antiproliferative effect; synergizes with albendazole sulfoxide (Loos et al., 2022).	*In vitro*	Better therapeutic effect

## 4 Anti-tumor drugs

The mechanism of antitumor drugs in the treatment of echinococcosis mainly involves inhibiting cell proliferation, inducing apoptosis, suppressing angiogenesis, interfering with kinase signaling pathways, disrupting metabolic reprogramming, and targeting parasite stem cells. Since the 1970s, researchers have begun to explore the application of antitumor drugs in the treatment of echinococcosis (Lubinsky, 1969). Some cytostatic agents can interfere with the cell cycle and metabolic processes of parasites, thereby inhibiting their growth (Pensel et al., 2014; Hübner et al., 2010).

### 4.1 Tyrosine kinase inhibitors (TKIs)

Sorafenib, As a multi-kinase inhibitor, the anti-echinococcosis mechanism of sorafenib mainly involves inhibiting MEK/ERK, two homologous proteins in The Mitogen-activated Protein Kinase (MAPK) cascade pathway of Echinococcus. It has shown significant anti-echinococcosis effects both *in vitro* and in animal models, and exhibits a strong killing effect especially on protoscoleces and germinal layer cells (Siles-Lucas, 2021). Zhang et al. (2018) utilized this mechanism by adding sorafenib tosylate, PD184352, or U0126-ethanol to the *in vitro* culture system of protoscoleces or cysts of *Echinococcus granulosus*. By inhibiting the phosphorylation of EgMKKs and EgERK, they observed a cytolysis inhibitory effect. The study showed that the protoscoleces treated with sorafenib tosylate and U0126-ethanol were completely inactivated.

In conclusion, the MAPK cascade pathway has been identified as a new target for drug development, and its inhibitors can effectively inhibit *Echinococcus granulosus in vitro*. However, to translate these findings into *in vivo* efficacy, further optimization of the therapeutic regimens for sorafenib tosylate or other kinase inhibitors is required.

Imatinib: The larval stage of *Echinococcus* also relies on certain kinase signaling pathways for cell proliferation and survival, and TKIs can inhibit these pathways to directly suppress the proliferation of the parasite (Hemer and Brehm, 2012). Hemer and Brehm (2012) reported in their study that imatinib can efficiently kill *Echinococcus* stem cells, larval vesicles, and protoscoleces *in vitro*. At a concentration as low as 10 μM, it can significantly inhibit the differentiation of parasite stem cells into larval vesicles, making it a promising new alternative to benzimidazoles in the chemotherapy of echinococcosis. In addition, imatinib can also be used as a lead compound to develop derivatives with stronger antiparasitic activity, and the *Echinococcus ABL* kinase sequence identified in this study will provide an important basis for the identification of such compounds.

### 4.2 Metallomatrix protease inhibitors

Bortezomib is used for the treatment of multiple myeloma and mantle cell lymphoma (Bross et al., 2004; Kane et al., 2007). It shows significant efficacy in *in vitro* experiments, but fails to achieve the expected effect in mice *in vivo* (Stadelmann et al., 2014). Stadelmann et al. (2014) also showed in animal models that at a dose of 0.5 mg/kg bortezomib combined with ABZ, the average weight of the parasite was reduced by 2 g, demonstrating that bortezomib can serve as a drug target for *Echinococcus multilocularis*, though its actual efficacy requires further verification.

### 4.3 Other anti-tumor drugs

Bevacizumab, as an anti-Vascular Endothelial Growth Factor humanized monoclonal antibody, exerts its mechanism of action by specifically blocking the vascular endothelial growth factor signaling pathway to inhibit pathological angiogenesis (Chen and Wang, 2022). Chen et al. (2025) pointed out in their research on hepatocellular carcinoma that the activation of the VEGF/VEGFR pathway is the core mechanism underlying abnormal proliferation of tumor blood vessels, and this mechanism is similar to the formation of the vascular network around hepatic echinococcal vesicles. In hepatic echinococcosis, *Echinococcus granulosus* infection can stimulate excessive secretion of VEGF in the host’s liver tissue, promoting the formation of a rich vascular network around the vesicles to meet the nutritional needs of the parasite. Current studies have initially revealed the potential value of bevacizumab in the treatment of parasitic diseases. However, the interaction mechanism between parasite-derived angiogenic factors (such as VEGF analogs secreted by *Echinococcus granulosus*) and host pathways has not yet been clarified, resulting in insufficient precision of targeted therapy. Meanwhile, there is a lack of an angiogenesis evaluation system that reflects the dynamic characteristics of parasitic infections, and whether existing oncological markers (such as circulating VEGF levels) can predict therapeutic efficacy remains controversial.

The latest research indicates (Gao et al., 2024) that tyrosine kinase inhibitors (TKIs) targeting RTKs, such as lenvatinib for vascular endothelial growth factor (VEGF)-initiated pathological angiogenesis in solid tumors; and drugs like gefitinib and cetuximab, which target the epidermal growth factor receptor (EGFR) signaling pathway in non-small cell lung cancer and metastatic colorectal cancer, can inhibit the development of polycystic echinococcus larvae *in vitro* and/or *in vivo* experiments and play a significant role in the progression of parasitic diseases such as echinococcosis.

However, antitumor drugs themselves have high toxicity, and their targets and mechanisms of action in echinococcosis have not been fully clarified. More clinical studies are still needed to further verify the mechanism of action. Progress in the Development of Anticancer Drugs as Shown in Table 2.

**TABLE 2:** Progress in the development of anti-tumor drugs.

Drug category	Representative drugs	Main mechanism of action	*In vitro or* *in vivo*	Treatment effect
Tyrosine kinase inhibitors (TKIs)	Sorafenib	Inhibits MEK/ERK in Echinococcus MAPK cascade pathway; blocks phosphorylation of EgMKKs and EgERK (Siles-Lucas, 2021).	*In vitro* (Siles-Lucas, 2021; Zhang et al., 2018)	Better therapeutic effect
	Imatinib	Inhibits parasite kinase signaling pathways and targets Echinococcus ABL kinase (Hemer and Brehm, 2012).	*In vitro* (Hemer and Brehm, 2012)	Better therapeutic effect
Metallomatrix protease inhibitors	Bortezomib + ABZ	Targets *Echinococcus* proteasome; synergizes with ABZ (Stadelmann et al., 2014)	*In vitro*	Has a certain effect
	Bevacizumab	Blocks VEGF/VEGFR pathway; inhibits pathological angiogenesis induced by parasite infection (Chen and Wang, 2022)	​	Better therapeutic effect

## 5 Research on the active components of traditional Chinese and Tibetan medicines

### 5.1 Artemisinin

It is a sesquiterpene lactone natural product isolated from Artemisia annua, a plant of the Asteraceae family. The main mechanism of artemisinin derivatives involves iron ion-mediated free radical reactions, which increase the content of Reactive Oxygen Species (ROS) in parasites. Their molecular structure contains a characteristic endoperoxide bridge, which is rapidly oxidized, thereby acting on the cofactors of the Antioxidant Glutathione (GSH)/thioredoxin system in the parasite. This results in the inability of GSH to control the production of *ROS* in the parasite, leading to the death of the parasite (Pharmacy, 2018). Ma et al. (2020) conducted *in vitro* experiments by co-culturing dihydroartemisinin at different concentrations with *Echinococcus granulosus* protoscoleces for 8 days. Among them, all protoscoleces died after 5 days of treatment with 200 μmol/L dihydroartemisinin. In addition, under the treatment of dihydroartemisinin at different concentrations, the activity of caspase-3 showed a time- and dose-dependent increasing trend within 2–4 days. The experimental results indicate that dihydroartemisinin can effectively promote the apoptosis of *Echinococcus granulosus* protoscolex cells by activating the endoplasmic reticulum *stress/caspase-3 signaling* pathway, suggesting its research potential. When reviewing the development history of anti-infective drugs, Wang et al. (2022) emphasized that the success of artemisinin-based drugs stems from the systematic exploration of Traditional Chinese Medicine (TCM). This research paradigm provides methodological guidance for discovering new anti-echinococcal compounds from natural products. However, the efficacy of artemisinin when used alone is inferior to that of albendazole. Furthermore, more in-depth research on its anti-echinococcosis effect has not been conducted in previous studies. Although these studies have confirmed that such drugs possess anti-echinococcosis activity, their mechanism of action remains unelucidated (Pharmacy, 2018).

### 5.2 Sophora moorcroftiana

As a characteristic Tibetan medicine component on the Qinghai-Tibet Plateau, the anti-echinococcal mechanism of Sophora moorcroftiana alkaloids mainly involves increasing the proportion of CD4^+^ T cells to enhance the immune response, downregulating the expression of programmed death receptor-1 (a negative regulatory factor on the T cell surface), and regulating the levels of interferon-γ (IFN-γ) and interleukin-4 (IL-4) to promote immune function (Luo, 2017). A study by Zhang (2022) showed that after co-culturing Sophora moorcroftiana alkaloid E2-a at different concentrations with Echinococcus multilocularis cysts for 5 days, varying degrees of damage were caused to the cysts. This finding not only verifies the potential medicinal value of Sophora moorcroftiana alkaloids but also provides a scientific basis for the development of new antiparasitic drugs. However, the composition of Sophora moorcroftiana alkaloids is complex, and systematic studies on the main active components and their targets have not yet been conducted. Therefore, the therapeutic effect on hepatic echinococcosis still needs to be further confirmed by a large number of clinical trials.

### 5.3 Lycorine

Lycorine is an alkaloid natural product derived from the bulbs of perennial herbaceous plants in the Amaryllidaceae family. It exhibits significant activities such as antiparasitic and antitumor effects, and its mechanism of anti-inflammatory and immunomodulatory actions shows unique value in the treatment of hepatic echinococcosis (Roy et al., 2018). Li et al. (2020) confirmed through animal experiments that lycorine can significantly increase the proportions of CD4^+^ T lymphocytes and NK cells in the spleens of mice infected with Toxoplasma gondii. Meanwhile, it reduces the levels of pro-inflammatory factors IFN-γ, IL-1β, and IL-9, and enhances the expression of the anti-inflammatory factor IL-4. This bidirectional immunomodulatory effect helps control parasitic infections and alleviate immunopathological damage. A study on Sophora moorcroftiana alkaloids by Du et al. (2023) showed that plant-derived alkaloids can inhibit the activity of *Echinococcus protoscoleces* by inducing the caspase-3-dependent apoptotic pathway. As an alkaloid with a similar structure, lycorine may exhibit a similar direct parasiticidal effect. A study by Yang et al. (2020) showed that lycorine, a component of traditional Chinese medicine, can effectively kill *Echinococcus granulosus*, with the effect showing a concentration-dependent relationship. Its efficacy is significantly superior to that of albendazole, a traditional drug, and it may produce a synergistic effect with albendazole. The experimental results indicated that lycorine has a significant inhibitory effect on *Echinococcus* and significantly enhances the therapeutic effect. However, most studies are still limited to *in vitro* experiments and animal experiments, and its precise targets and complete mechanism of action in the human body remain unclear, which limits its clinical application.

### 5.4 Trigonelline

Trigonelline is a polar hydrophilic alkaloid derived from medicinal plants (including Chinese herbal plants). It possesses a variety of pharmacological activities, including antioxidant, antibacterial, hypoglycemic, antitumor effects, as well as protective effects on liver and kidney functions (Zhang et al., 2021; Mohamadi et al., 2018). Its mechanism mainly involves killing protoscoleces by activating cysteine-aspartic acid protease-3 (caspase-3) to induce apoptosis, and inhibiting the nuclear factor erythroid 2-related factor 2 (Nrf2) signaling pathway to regulate the activities of Reactive Oxygen Species (ROS), Heme Oxygenase-1 (HO-1), and NADH dehydrogenase quinone-1 (NQO-1) in *Echinococcus granulosus* protoscoleces (Qin et al., 2017). Chen et al. (2023) showed through *in vitro* experiments on mice that 250 μmol/L trigonelline could cause the death of all *Echinococcus granulosus* protoscoleces after 12 days of *in vitro* treatment, confirming the role of trigonelline in anti-echinococcosis.

### 5.5 Other potential active components of traditional Chinese medicine and Tibetan medicine

In addition to the aforementioned components, various active ingredients of traditional Chinese medicine have shown anti-echinococcal potential. Harmaline is a β-pyridine alkaloid extracted from the seeds of Peganum harmala, which exhibits strong antiparasitic activity (Zhang et al., 2020). Recent studies have found that it can induce DNA damage in protoscoleces by activating the ATM-P53-Topo2a signaling pathway. At a concentration of 25 μg/mL, it can cause the death of all protoscoleces after 3 days of action, and its efficacy is superior to that of albendazole (Lu et al., 2021). Curcumin, on the other hand, relies on its excellent anti-inflammatory, antioxidant, and antifibrotic properties (Shu and Pan, 2007) shows application prospects in alleviating liver tissue damage and fibrosis around echinococcal cysts. In addition, paclitaxel has demonstrated excellent therapeutic effects on various tumors (Sharifi-Rad et al., 2021). Pensel et al. (2014) confirmed through *in vitro* and *in vivo* experiments that it can cause the death and structural damage of protoscoleces (such as vacuolation and loss of microtriches). For isolated parasite cells, it can inhibit their proliferation and induce apoptotic/necrotic morphologies such as cell atrophy and surface blebbing, thereby exerting an anti-echinococcosis effect. These components together constitute a natural molecular library for the discovery of anti-echinococcal drugs, which is of great exploration value.

Although traditional Chinese medicine holds great potential in future research, it still faces significant challenges in the treatment of echinococcosis (hydatid disease) due to key issues including complex active components, unclear mechanisms of action, difficulty in quality control, and lack of standardized clinical efficacy evaluation. Research and Development Progress of Traditional Chinese Medicine as Shown in Table 3.

**TABLE 3:** Research and development progress in traditional Chinese medicine.

Drug category	Representative drugs	Main mechanism of action	*In vitro or* *in vivo*	Treatment effect
Traditional Chinese medicine components	Dihydroartemisinin	Dihydroartemisinin inhibits apoptosis in the protostominal cells of Echinococcus granulosus by activating the endoplasmic reticulum stress/caspase-3 signaling pathway (Ma et al., 2020).	*In vitro* (Ma et al., 2020)	Artemisinin is less effective than albendazole (Wang et al., 2022).
	Sophora	Increases CD4^+^ T cell proportion; downregulates PD-1 expression; regulates IFN-γ and IL-4 levels (Luo, 2017).	*In vitro*	Has a certain effect
	Lycorine	Bidirectional immunomodulation; may induce caspase-3-dependent apoptosis of protoscoleces (Du et al., 2023).	*In vitro* *or* clinical trial, animal experiments (Li et al., 2020; Yang et al., 2020).	Better therapeutic effect
	Trigonelline	Activates caspase-3 to induce protoscolex apoptosis; inhibits Nrf2 signaling pathway (Qin et al., 2017).	*In vitro* (Chen et al., 2023)	Better therapeutic effect
	Harmaline	Activates ATM-P53-Topo2a signaling pathway; induces protoscolex DNA damage (Lu et al., 2021).	*In vitro*	Better therapeutic effect
	Paclitaxel	Induces apoptosis/necrosis of parasite cells; causes structural damage (vacuolation, microtriche loss) (Pensel et al., 2014).	*In vivo* (Pensel et al., 2014)	Better therapeutic effect

## 6 Summary and outlook

Currently, the drug treatment of hepatic echinococcosis still faces challenges such as limited efficacy, long treatment course, many adverse reactions, poor patient compliance, and potential drug resistance. Although benzimidazole drugs, such as albendazole, have been widely used in clinical practice, their low solubility and poor bioavailability have limited the drug efficacy to a certain extent. To further improve the therapeutic effect of these drugs, in recent years, through structural modification, nano-delivery systems, combination therapy strategies, and the exploration of natural drugs and active components of traditional Chinese medicine, the research on anti-echinococcosis drugs has shown a diversified and precise development trend.

Future research on anti-echinococcosis should focus on five directions: First, using multi-omics technology to accurately identify targets, deeply analyze the molecular mechanism of parasite survival and pathogenicity, and provide a precise target basis for drug development; second, developing intelligent nano-delivery systems, optimizing the biocompatibility and targeting of nanocarriers, improving drug efficacy and reducing toxic and side effects; third, verifying combination therapies through high-quality clinical studies, clarifying the optimal combination scheme, administration timing and dosage, and providing reliable clinical evidence for combined medication; fourth, exploring the value of traditional drugs with modern technology, separating and purifying active components from traditional Chinese and Tibetan medicines, clarifying their mechanisms of action and targets, and realizing the modernization and standardization of traditional drugs; fifth, strengthening interdisciplinary and international cooperation, integrating resources from pharmacology, molecular biology, nanotechnology, clinical medicine and other fields, and accelerating the transformation of basic research results into clinical applications. Ultimately achieving effective control and elimination of hepatic echinococcosis.

In conclusion, with the deepening of basic research and the advancement of technical means, it is expected that safer, more effective, and shorter-course new anti-echinococcosis drugs will be developed in the future, providing strong support for the effective control and elimination of *Echinococcosis*.
